# Genetic Variability in eIF2*α* Gene Is Associated with Islet *β*-Cell Function in the Development of Diabetes in a Chinese Han Population

**DOI:** 10.1155/2018/6590532

**Published:** 2018-02-20

**Authors:** Nan Gu, Xiaowei Ma, Jianwei Zhang, Mengmeng Jin, Nan Feng, Ruifen Deng, Ge Bai, Hong Zhang, Xiaohui Guo

**Affiliations:** Endocrinology Department, Peking University First Hospital, Beijing, China

## Abstract

**Aims:**

Protein kinase-like endoplasmic reticulum kinase (PERK)/eukaryotic translation initiation factor 2 alpha (*eIF2α*) pathway mutations lead to failure of *β*-cell function. The aim of this article was to assess the association between *eIF2α* and the risk of glucose metabolism abnormalities.

**Methods:**

Two *eIF2α* SNPs (rs9840992 T>C and rs13072593 A>G) were selected based on CHB data from HapMap, and 1466 unrelated nondiabetes individuals were genotyped. All subjects were examined by the 75 g oral glucose tolerance test, and 733 participated in a subsequent insulin release test. Various indicators of insulin resistance and islet *β*-cell function were examined.

**Results:**

There were no significant differences in genotype distribution and allele frequency between the prediabetes and controls. CC genotype carriers at rs9840992 showed higher insulin levels at 120 min after a 75 g glucose load than noncarriers. Also, CC homozygotes had higher ΔI30/ΔG30 and ΔI120/ΔG120 than noncarriers, even after adjusting for insulin resistance. CC homozygotes had greater AUCi values than noncarriers. Subjects aged ≥ 65 yrs, those with a BMI ≥ 24 kg/m^2^ and those carrying the rs9840992 risk allele, had a 2.5-fold higher risk of glucose abnormalities than subjects who had none of these risk factors.

**Conclusion:**

The *eIF2α* polymorphism is associated with islet *β*-cell function in a Chinese population.

## 1. Introduction

Pancreatic *β*-cell dysfunction is a key factor in the development and progression of type 2 diabetes. Prediabetes, which is characterized by impaired fasting glucose and/or impaired glucose tolerance [1], pancreatic *β*-cell dysfunction, and insulin resistance are already in the early stages. Several genetic and environmental factors are likely to contribute to the progressive abnormality or even failure of pancreatic *β*-cell function, but the precise mechanism is still unknown. However, endoplasmic reticulum (ER) stress is a likely contributing factor.

Many studies have demonstrated that the unfolded protein response (UPR), induced by the accumulation of unfolded or misfolded proteins in the ER, plays an important role in chronic metabolic diseases, such as insulin resistance and type 2 diabetes. *EIF2α* is part of the complex that recruits the initiator methionyl-tRNA to the ribosome and is phosphorylated by protein kinase-like ER kinase (PERK) [2]. The PERK/*eIF2α* pathway plays a key role in transcriptional control [3, 4]. We inferred that genetic variation in *eIF2α* might contribute to changes in pancreatic *β*-cell function or insulin sensitivity. To evaluate this hypothesis, we studied genetic variation in the *eIF2α* gene in a Chinese population with prediabetes.

## 2. Materials and Methods

### 2.1. Ethics Statement

The research ethics committee of Peking University First Hospital approved the study protocol. In agreement with the Declaration of Helsinki, all 1466 unrelated subjects provided informed consent to participate in this study.

### 2.2. Study Population

The Epidemiological Survey on Diabetes was conducted in Beijing from 2007 to 2009 and included three large communities. Subjects were recruited from 2007 to 2009. Prediabetes was defined by the 1999 World Health Organization criteria based on the oral glucose tolerance test (75 g OGTT), which stipulates fasting plasma glucose (FBG) values of between 6.1 mmol/L and 7.0 mmol/L, 2 h post-OGTT (PBG) levels of between 7.8 mmol/L and 11.1 mmol/L, or both. A total of 1466 unrelated Han Chinese subjects were included in the study, of which 845 were prediabetes and 621 were included as controls. Controls were ≥55 years old and had no family history of diabetes.

## 3. Methods

All subjects underwent a standardized clinical and laboratory evaluation as described previously [5]. Fasting blood was collected to measure glucose and lipid levels in the morning (Table 1). Genomic DNA was extracted from leukocytes using the salting-out method [6]. Homeostasis model assessments of insulin resistance (HOMA-IR) and *β*-cell function (HOMA-*β*) were calculated using the formulae described by Matthews et al. [7]. Fasting glucose, 2 h PBG, total cholesterol (TC), triglycerides (TG), high-density lipoprotein cholesterol (HDL-C), fasting insulin levels (INS0), post-OGTT30 insulin levels (INS30), and 2 h post-OGTT insulin levels (INS120) were measured as described in detail previously [5]. HOMA-IR, HOMA-*β*, and areas under the curve for insulin (AUCi) were calculated using the formulae described by Matthews et al. [7]. The function of *β*-cells was quantified as the ratio of the incremental insulin to glucose responses over the first 30 min (G30) and 120 min during the OGTT (ΔI30/ΔG30 = (INS30−INS0)/(G30−FBG) and ΔI120/ΔG120 = (INS120−INS0)/(PBG−FBG)).

## 4. Genotyping

The *eiF2α* gene is located at chromosome 3q25.1, spans 65 kb, and includes 14 exons [8]. Two haplotype-tagging SNPs (*r*^2^ < 0.8, MAF ≥ 0.05) were identified at the *eiF2α* locus from CHB data (Han Chinese from Beijing) obtained in HapMap phase II (R#27, http://www.hapmap.org). The SNPs rs9840992 (T>C) and rs13072593 (A>G) were chosen. Direct DNA sequencing was performed using the MassArray system (Sequenom iPLEX Assay, San Diego, CA, USA) [9].

## 5. Statistical Analysis

All statistical analyses were performed using the SPSS statistical package (version 13.0; SPSS, Chicago, IL, USA). Genotype distributions were used to assess departures from Hardy–Weinberg equilibrium at each polymorphic locus. Clinical and laboratory data are expressed as means ± standard deviation or as medians (lower-upper quartiles). Comparisons of the clinical and laboratory parameters between the prediabetes and control groups as well as between genotype groups were performed using unpaired Student's *t*-tests or *χ*^2^ tests. Associations between prediabetes and genotypes were analyzed using multiple logistic regressions, with adjustments for the following potential confounders: gender, age, BMI, and waist-hip ratio. As a descriptive measure of the association between genotypes and outcomes, *p* < 0.05 was considered statistically significant and odds ratios (ORs) were calculated with 95% confidence intervals (CIs). Linkage disequilibrium, haplotype block structure (using a CI algorithm [10]), and haplotype analyses were performed using HaploView 4.1 (http://www.broadinstitute.org/haploview/haploview) [11]. Haplotype frequencies were estimated using an accelerated expectation-maximization algorithm [12]. Bonferroni correction was used to correct for multiple comparisons. Power and sample size calculation (version 3.1.2, 2014) was used for power calculation [13]. Statistical power was 0.826.

## 6. Results

The genotype distributions were in Hardy–Weinberg equilibrium at both loci (*p* > 0.01). People with prediabetes were younger and had higher BMIs and waist-hip ratios, higher TG levels, and lower HDL-C levels than control individuals (Table 1).

The genotype distribution and allele frequencies were not significantly different between the prediabetes and control groups (Table 2). SNPs rs9840992 and rs13072593 were distributed in one linkage disequilibrium (LD) block. We also compared the haplotype distributions between prediabetes and controls. Haplotype (AT, GC, and AC) distributions between prediabetes and controls showed no statistical significant difference.

We measured plasma insulin following 75 g OGTT in 748 of the 1466 total individuals. The carriers of genotype CC at rs9840992 had higher insulin levels at 120 min after the 75 g glucose load (INS120) than noncarriers (49.86 (30.38–79.61) versus 44.32 (26.42–70.75), *p* = 0.038). We also found that CC homozygotes had higher ΔI30/ΔG30 (10.82 (6.25–19.35) versus 8.97 (5.35–16.49), *p* = 0.008) and ΔI120/ΔG120 values (23.02 (13.47–43.21) versus 19.38 (10.45–36.57), *p* = 0.017) compared to those of noncarriers, and the differences were still significant after adjusting for insulin resistance (*p*′ = 0.004 and *p*′ = 0.006, resp.). CC homozygotes had greater AUCi values than those of noncarriers (78.60 (54.42–116.29) versus 69.00 (47.51–108.07), *p* = 0.023; adjusted *p*′ = 3.7 × 10^−4^ for insulin resistance) (Table 3).

**Figure 1:**
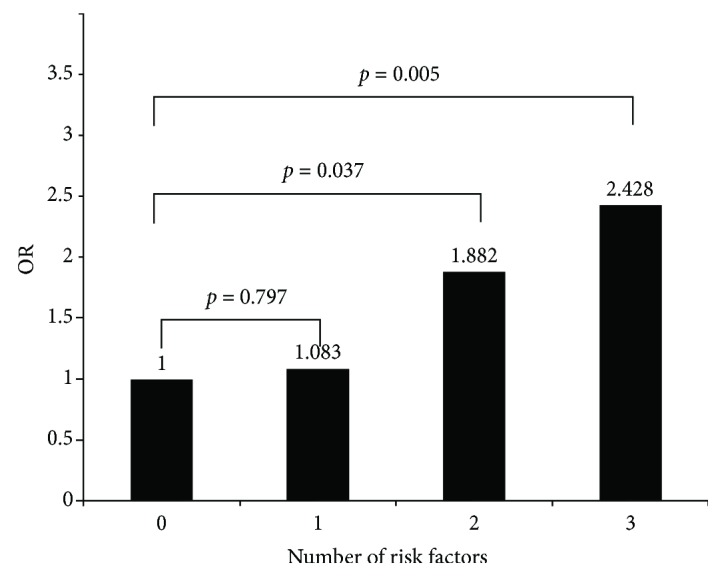
Number of risk factors and the relative risk of prediabetes. Risk factors: age ≥ 65 yr; BMI ≥ 24 kg/m^2^; carrying the T allele of SNP rs9840992.

When we included age ≥ 65 yr, BMI ≥ 24 kg/m^2^, and carrying the risk allele T for the SNP rs9840992 as risk factors of prediabetes, we observed that the more factors an individual carried, the higher the risk for prediabetes (*p* ≤ 0.001). Individuals who had two risk factors had an 80% higher risk (OR = 1.882, *p* = 0.0.037) than individuals without risk factors, and subjects who had all three risk factors had an approximately 2.5-fold greater risk (OR = 2.428, *p* = 0.005) compared with individuals who had none of the three factors (Figure 1).

## 7. Discussion

The ER is a complex membranous network in eukaryotic cells and is the site for the synthesis, folding, assembly, and posttranslational modification of proteins. It includes a highly conserved system of proteins that facilitates protein folding and processing and protects cells from the toxic effects of unfolded protein accumulation (i.e., ER stress). When these functions fail, the UPR is activated by the accumulation of unfolded proteins, and the apoptosis process is initiated [14–16]. The UPR pathway is particularly important in adipocytes, hepatocytes, and pancreatic *β*-cells [17]. There are three ER transmembrane sensors of unfolded proteins in murine cells: activating transcription factor 6*α*, inositol-requiring 1*α*, and the PERK/*eIF2α* pathway. Many studies have demonstrated that the PERK/*eIF2α* pathway is related to pancreatic *β*-cell function [16, 18]. *EIF2α* can also upregulate the expression of ATF4 and CHOP, leading to the apoptosis of islet *β*-cells [17].

In this study, we observed subjects with prediabetes to determine whether genetic variation in *eIF2α* contributes to the very early stage of pancreatic *β*-cell dysfunction. Interestingly, we observed an association between the SNP rs9840992 and islet *β*-cell function, but not prediabetes, in the Chinese Han population. To confirm this association, we used various parameters to evaluate islet *β*-cell function and obtained consistent results. Carriers of the major allele T had lower insulin levels, as estimated by ΔI30/ΔG30 (*p* = 0.008) and ΔI120/ΔG120 values (*p* = 0.017), than noncarriers, even after adjusting for insulin resistance (*p*′ = 0.004 and *p*′ = 0.006, resp.). Additionally, individuals carrying the T allele had lower AUCi values than noncarriers (*p* = 0.023), and this difference was highly significant, even after adjusting for insulin resistance (*p*′ = 3.7 × 10^−4^).

When we analyzed age ≥ 65 yr, BMI ≥ 24 kg/m^2^, and carrying the risk allele T of SNP rs9840992 as risk factors of prediabetes in a cumulative risk analysis, we found that the odds of glucose homeostasis disturbance were related to the number of the risk factors; that is, a higher number of factors was associated with an increased risk for prediabetes. Subjects who had all three risk factors had a risk that was 1.5 times higher than that of subjects who had none of the factors (*p* = 0.005). Several studies have confirmed that aging and obesity are risk factors for diabetes owing to their associations with *β*-cell dysfunction. Therefore, age, BMI, and genetic variation at rs9840992 each play a role in pancreatic *β*-cell function and thereby contribute to the cumulative susceptibility to diabetes. Our results suggest that individuals who carry the risk allele at this locus may benefit from early intervention to reduce body weight, which can significantly reduce the incidence of prediabetes and even diabetes.

The SNP rs9840992 is located in an intronic region. The mechanisms by which genetic variation in introns affects *β*-cell function are unknown. However, Zheng et al. [19] showed that intronic SNPs influence gene expression. Therefore, we postulate that rs9840992 modifies the mRNA and protein expression levels of *eIF2α*, and these changes may contribute to the modulation of islet *β*-cell apoptosis and insulin secretion. However, it is more likely that the SNP is in strong linkage disequilibrium with other SNPs that have biological effects.

No studies have examined *eIF2α* in prediabetes or type 2 diabetes patients. However, this study had some limitations. The relatively small sample size might bias the results. In addition, functional studies are necessary. Our findings should be replicated in other populations, and the SNPs should be confirmed as predictive genetic markers in prospective studies.

## 8. Conclusion

Our findings suggest that genetic variation at the *eIF2α* locus is associated with islet *β*-cell function in a Chinese population.

## Figures and Tables

**Table 1:** Clinical characteristics of cases and controls.

	Controls (*n* = 621)	p-DM (*n* = 845)	*p* value
Gender (M/F)	240/381	291/554	0.099
Age (y)	65.91 ± 7.07	61.75 ± 11.3	≤0.001
BMI (kg/m^2^)	25.11 ± 3.43	26.53 ± 3.77	≤0.001
WHR	0.87 ± 0.06	0.89 ± 0.31	0.041
FPG (mmol/L)	5.26 ± 0.42	5.72 ± 0.57	≤0.001
PBG-2h (mmol/L)	6.01 ± 1.07	8.57 ± 1.33	≤0.001
TC (mmol/L)	5.24 ± 1.00	5.28 ± 1.11	0.523
TG (mmol/L)	1.54 ± 0.99	1.86 ± 1.28	≤0.001
HDL-C (mmol/L)	1.35 ± 0.33	1.29 ± 0.35	0.005
LDL-C (mmol/L)	3.23 ± 0.94	3.25 ± 1.50	0.732

p-DM: prediabetes; BMI: body mass index; WHR: waist-to-hip ratio; FBG: fasting blood glucose; PBG-2h, post-OGTT 2-hour blood glucose; TC: total cholesterol; TG: triglycerides; HDL-C: high-density lipoprotein; LDL-C: low-density lipoprotein.

**Table 2:** Association between prediabetes and SNP genotypes at the *eIF2α* locus.

SNP genotypes	Control (*n* = 621)	p-DM (*n* = 845)	OR	95% CI	*p*	OR′	95% CI′	*p*′
rs9840992								
CC	152	194	0.950	0.744–1.212	0.678	0.917	0.710–1.184	0.507
TC/TT	468	629	1	—	—	—	—	—
rs13072593								
GG	105	142	0.975	0.739–1.286	0.857	1.007	0.754–1.346	0.962
AG/AA	515	679	1	—	—	—	—	—

SNPs: single nucleotide polymorphisms; p-DM: prediabetes. OR′, adjusted for gender, age, BMI, and WHR. *p*′, adjusted for gender, age, BMI, and WHR.

**Table 3:** Association between islet *β*-cell function and rs9840992 genotype in *eIF2α.*

	CC (*n* = 175)	TC/TT (*n* = 558)	*p* value
FBG (mmol/L)	5.51 ± 0.55	5.54 ± 0.57	0.376
PBG30′ (mmol/L)	9.35 ± 1.78	9.54 ± 1.88	0.201
PBG-2h (mmol/L)	7.50 ± 1.67	7.55 ± 1.76	0.626
INS0′ (mU/L)	7.52 (5.98–9.57)	7.54 (5.36–10.58)	0.656
INS30′ (mU/L)	49.31 (28.97–70.92)	41.23 (26.71–68.05)	0.301
INS120′ (mU/L)	49.86 (30.38–79.61)	44.32 (26.42–70.75)	0.038
HOMA-*β*	76.00 (57.03–103.79)	76.60 (53.72–103.25)	0.565
HOMA-IR	1.83 (1.41–2.42)	1.84 (1.28–2.61)	0.440
ΔI30/ΔG30	10.82 (6.25–19.35)	8.97 (5.35–16.49)	0.008
ΔI30/ΔG30/IR	5.87 (3.59–10.33)	4.88 (2.88–8.24)	0.004
ΔI120/ΔG120	23.02 (13.47–43.21)	19.38 (10.45–36.57)	0.017
ΔI120/ΔG120/IR	12.11 (8.10–21.93)	10.03 (5.84–18.58)	0.006
AUCi (mU/L^∗^h)	78.60 (54.42–116.29)	69.00 (47.51–108.07)	0.023
AUCi/IR (mU/L^∗^h)	44.08 (32.50–60.36)	37.50 (28.50-51.19)	3.7 × 10^−4^

FBG: fasting blood glucose; PBG30′: post-OGTT 30′ glucose; PBG-2h: post-OGTT 2-hour blood glucose; INS0′: fasting insulin levels; INS30′: post-OGTT30′ insulin levels; INS120′: 2-hour post-OGTT insulin levels; HOMA-*β*: *β*-cell function; HOMA-IR: homeostasis model assessments of insulin resistance; ΔI30/ΔG30/IR: the ratio of ΔI30/ΔG30 to HOMA-IR; ΔI120/ΔG120/IR: the ratio of ΔI120/ΔG120 to HOMA-IR; AUCi: area under the curve for insulin; AUCi/IR: the ratio of AUCi to HOMA-IR.
